# MyomirDB: A unified database and server platform for muscle atrophy myomiRs, coregulatory networks and regulons

**DOI:** 10.1038/s41598-020-65319-z

**Published:** 2020-05-25

**Authors:** Apoorv Gupta, Sukanya Srivastava, Geetha Suryakumar, Bhuvnesh Kumar, Pankaj Khurana

**Affiliations:** Defence Institute of Physiology and Allied Sciences (DIPAS), Defence R&D Organization (DRDO), Timarpur, Delhi India

**Keywords:** Genetic databases, Gene regulatory networks, Network topology

## Abstract

Muscular atrophy or muscle loss is a multifactorial clinical condition during many critical illnesses like cancer, cardiovascular diseases, diabetes, pulmonary diseases etc. leading to fatigue and weakness and contributes towards a decreased quality of life. The proportion of older adults (>65 y) in the overall population is also growing and aging is another important factor causing muscle loss. Some muscle miRNAs (myomiRs) and their target genes have even been proposed as potential diagnostic, therapeutic and predictive markers for muscular atrophy. MyomirDB (http://www.myomirdb.in/) is a unique resource that provides a comprehensive, curated, user- friendly and detailed compilation of various miRNA bio-molecular interactions; miRNA-Transcription Factor-Target Gene co-regulatory networks and ~8000 tripartite regulons associated with 247 myomiRs which have been experimentally validated to be associated with various muscular atrophy conditions. For each database entry, MyomirDB compiles source organism, muscle atrophic condition, experiment duration, its level of expression, fold change, tissue of expression, experimental validation, disease and drug association, tissue-specific expression level, Gene Ontology and KEGG pathway associations. The web resource is a unique server platform which uses in-house scripts to construct miRNA-Transcription Factor-Target Gene co-regulatory networks and extract tri-partite regulons also called Feed Forward Loops. These unique features helps to offer mechanistic insights in disease pathology. Hence, MyomirDB is a unique platform for researchers working in this area to explore, fetch, compare and analyse atrophy associated miRNAs, their co-regulatory networks and FFL regulons.

## Introduction

Muscle comprises approximately 40% of body weight and is very important for voluntary and involuntary actions performed by the body^1^. The growth and regeneration of any muscular tissue is a highly organized process and known as myogenesis^2^. It helps to maintain muscle mass. On the other side, the loss of muscle mass is generally termed as muscle atrophy. It is a natural process where the unused or inactive muscle in the body starts to waste away and leads to a loss in muscle mass and eventually physical strength. Muscle atrophy is an important clinical problem which limits the mobility of a person and a decline in physical performance. Many causes can lead to muscle atrophy e.g. aging, hypoxia, alcohol-associated myopathy, injuries such as wounds and burns, stroke, long-term corticosteroid therapy, diabetes myopathy, cancers, etc. Muscle atrophy in-fact increases the risk of morbidity/mortality in primary muscle diseases and secondary muscle disorders.

In muscles, protein turnover rate is regulated by protein synthesis and protein degradation and the imbalance leads to either muscular hypertrophy or atrophy. Positive regulation of several signaling pathways during myogenesis can increase muscle mass. These include Insulin-like growth factor signaling pathway, PI3K signaling, Akt signaling, etc. Whereas protein degradation pathways, the autophagy-lysosome systems, and the ubiquitin- proteasome are triggered during muscle atrophy and this leads to the loss of muscle mass^3^. Recent, pieces of evidence have indicated that miRNAs regulate the growth, regeneration, and metabolism of muscles^2^. MyomiRs is a special term coined to refer to a special class of miRNAs that regulate the gene expression in muscles. Currently, the studies on myomiRs miR-1, miR- 133 and miR-206 have gained tremendous attention in exploring miRNA functions in muscle atrophy^4,5^. Activation of myomiRs miR-1, miR-133, miR-206, miR-125b were found to promote PI3K signaling and Akt signaling by regulating their participating genes-expression- level that in-turn increases muscle mass^1^. These pathways promote muscle growth by increasing protein synthesis and decreasing protein degradation. Additionally, miRNAs like miR-181 are known to regulate myoblast differentiation^6^. Expression of miR-542-5p and miR- 424-5p were found to be significantly increased in patients with ICU-acquired amyotrophic lateral sclerosis and muscle atrophy^7–9^. The increase in myostatin level promotes muscle wasting, and some miRNAs e.g. miR-499, -208b, and -23a that downregulate its activity have been proposed as a treatment for muscle atrophy^1,10,11^. MiR-29b upregulation is associated with muscular atrophy and has been proposed as a therapeutic target to prevent atrophies^12,13^. Growing data from such research provides a comprehensive explanation for the underlying mechanisms during muscular atrophy in different pathological conditions.

Additionally miRNAs are themselves regulated by other regulatory molecules i.e. Transcription Factors (TFs). Transcription factors and miRNAs are known to regulate each other as well as their target genes; TFs effect miRNA expression, and miRNAs may repress TF expression. There are cases where TF and miRNA both regulate the expression of a Target Gene (TG). In the last decade, many reports hint that they may work in conjunction to influence the precise control of TG expression level. The complex interactions between miRNAs, TFs, and a common TG can be identified using tripartite motif commonly known as Feed‐Forward Loops (FFLs)^14,15^. FFL motif is composed of three molecular components: a TF, a miRNA and a TG in which either the TF or miRNA or both regulate each other expression and also of the target gene. The FFL motifs have been found to play vital roles in disease development, drug repurposing, and disease recurrence and hence can be used for the understanding of the underlying mechanism of disease initiation, progression, and recurrence. Recently, miRNA based FFLs are proposed as potential biomarkers for complex multifactorial disorders like myocardial infarct, human mesothelioma, cancer etc^16,17^. Analysis of miRNA-TF-TG co-regulatory networks in these diseases have found to successfully predict the disease pathology and recurrence^17–19^.

Currently there are several databases that contain information on muscle-related biomolecular studies e.g. NeuroMuscleDB- a database of genes associated with muscle development and neuromuscular diseases; SKmDB- a database of next-generation sequencing information of genes, miRNA, lncRNA in skeletal muscles; MuscleDB- a database which compiles RNA sequencing (RNA-seq) to profile global mRNA expression in a wide array of smooth, cardiac, and skeletal muscle tissues from mice and rats etc^20–22^. However, none of these databases cater to biomolecular responses during muscular degradation or atrophy. MyomirDB is a unique resource which compiles information on miRNA profile changes during muscular atrophy conditions in various pathological disorders and analyses these miRNAs by constructing miRNA-TF-TG coregulatory networks and identifying their FFLs to provide a unified view of the biomolecular responses during muscular atrophy. The datasets have been derived from the extensive data-mining and manual-curation of data from studies on miRNAs expressed in muscle atrophy using experimental techniques i.e. microarray, RNAseq, NGS, etc. The current version of MyomirDB contains 459 experimental associations of 247 unique miRNAs. For each database entry, details like source organism, atrophy condition, duration of experiment, level of expression, fold change, tissue of expression, type of experiment, association as a biomarker, reference paper are compiled. Additionally, functional correlations i.e. Gene Ontology and KEGG pathway are also identified and stored. To make MyomirDB more informative, miRNA-drug association, its expression in different diseases and tissues are also stored in the database. Various  bio-molecular interactions (miRNA:TG, miRNA:TF, TF:TG, TF:miRNA interactions) are stored and in-house scripts are used to construct miRNA-TF-TG co-regulatory networks which is displayed as a user interactive network and also available for download. Users can retrieve, compare, explore, analyze, and download the datasets through various search and browse options of the database.

## Results

### Web interface

MyomirDB is a freely available user-friendly resource to browse, retrieve, compare and analyse muscle-atrophy associated miRNAs. It is available online at http://www.myomirdb.in/ and requires no prior registration for access. It is a comprehensive, manually curated resource of miRNAs whose expression levels are experimentally validated to be associated with muscular atrophy. The database can be explored using the ‘Browse’ and ‘Search’ options.

**Figure 1:**
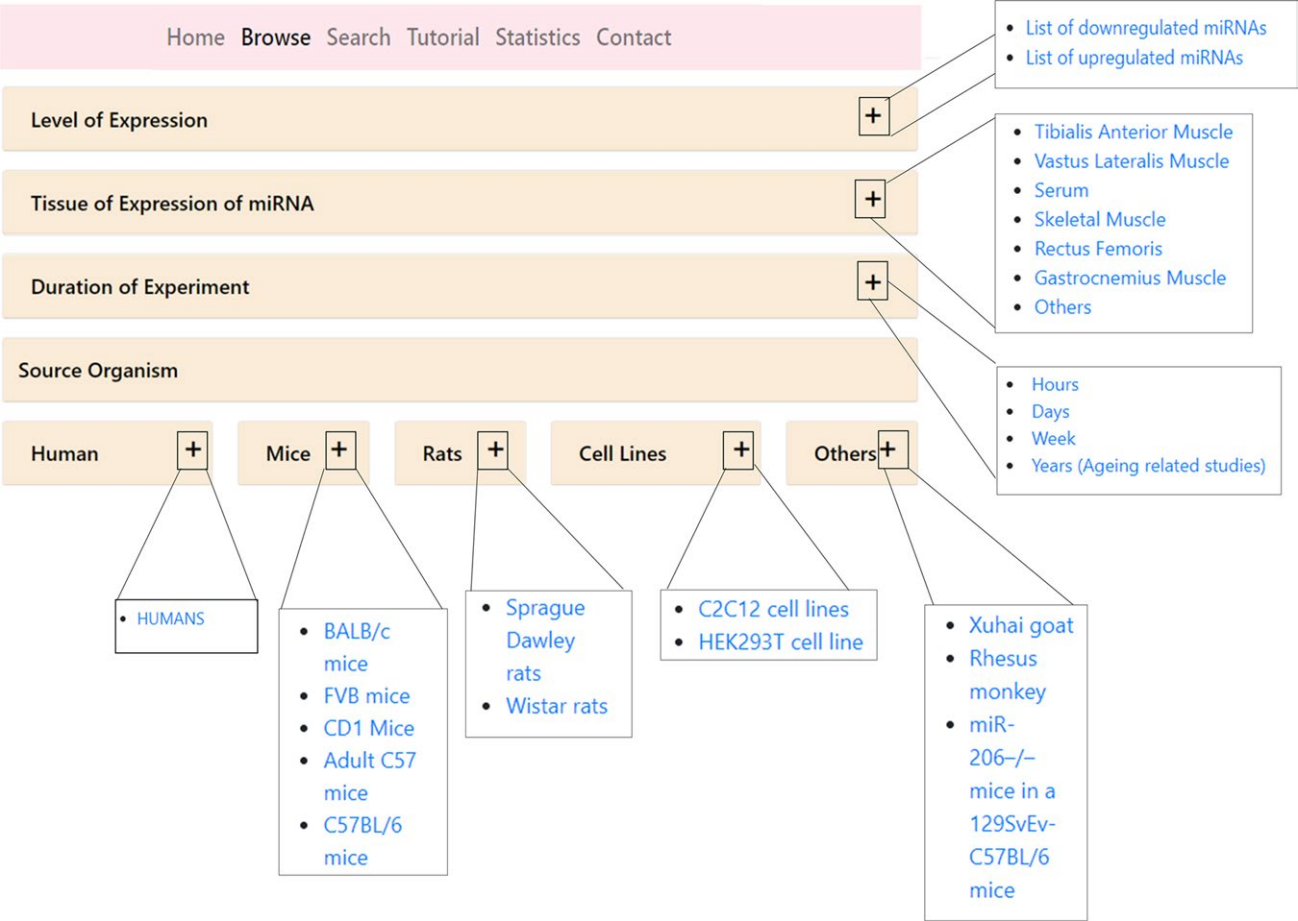
The figure shows the screenshot of the ‘Browse’ option. This option allow the users to browse atrophy miRNAs using different query filters. There are five queries filters namely ‘Level of expression’, ‘Tissue of expression’, ‘Duration of experiment’, ‘Source Organism’.

From the ‘Browse’ option, the data can be fetched using the filters which are designed as the drop-down menus that allow the users to select and retrieve the list of miRNAs (Fig. 1). These features allow the user to fetch information based on their research interests. Eg. Users interested in either upregulated/downregulated miRNAs in atrophy can use the first option and others interested in only human atrophy miRNAs may fetch the human associated studies using the ‘Source Organism’ option.

The filters are based on the level of expression (upregulated/ downregulated); tissue of expression (Tibialis Anterior Muscles/ Vastus Lateralis Muscle Biopsy/Serum/Skeletal muscles/Quadriceps/Rectus Femoris/Others); duration of experiment (hours, days, weeks and years aging-related studies); source organism (Human/Rat/ Mouse/Goat/Monkey).

**Figure 2:**
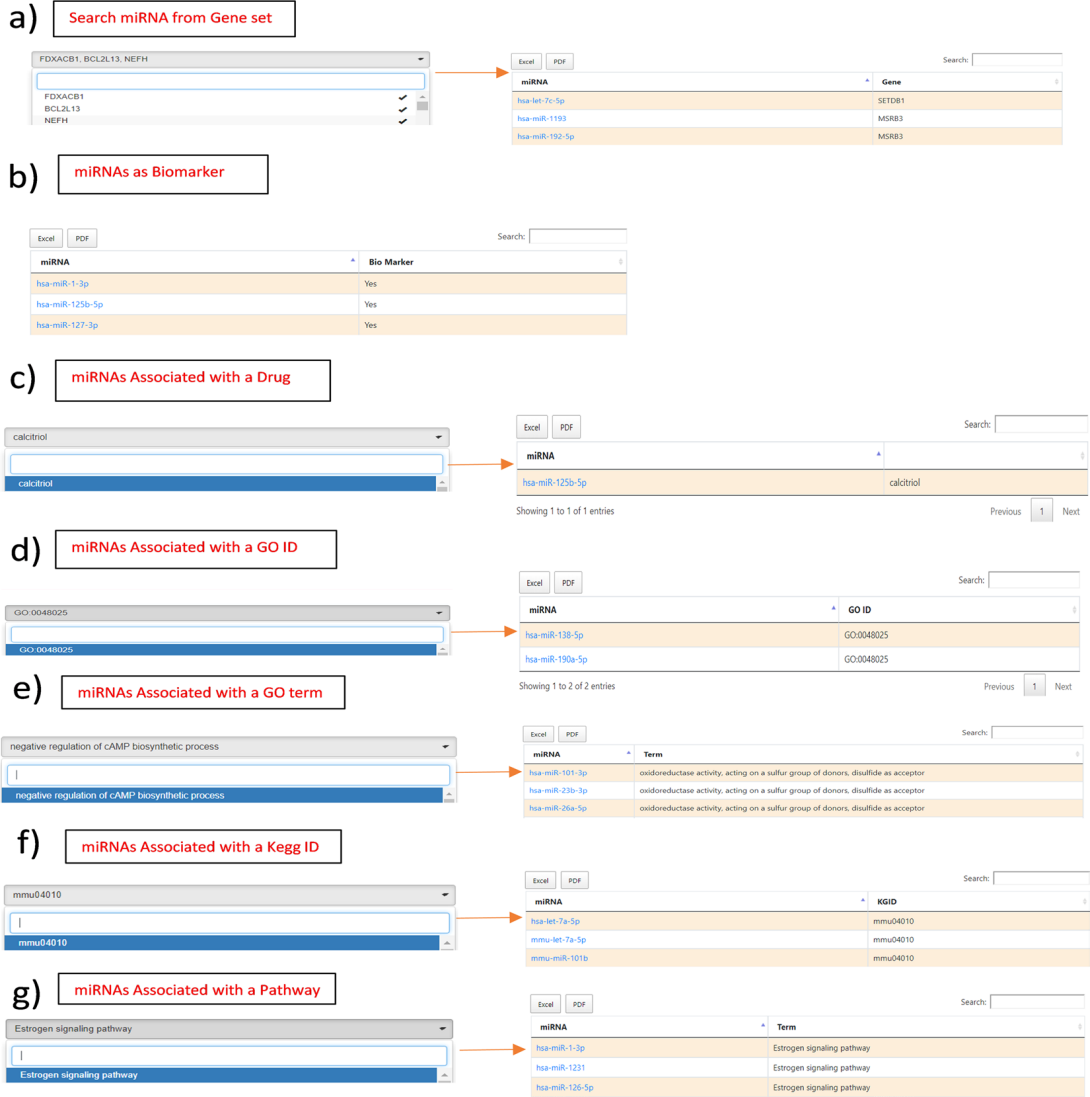
The web images shows the ‘Browse by association’ option. User can search myomiRs based on (**a**) user defined gene list, (**b**) association as biomarker, **(c**) association with drug, (**d**) association with GO ID (**e**) association with GO term (**f**) association with KEGG ID (**g**) association with KEGG pathway name.

In addition, the server offers “Browse by Association” option. This is useful to users if they have a gene-set or GO term of KEGG pathway of interest and wish to identify the associated atrophy myomiRs. This option offers a number of choices to browse E.g. “miRNAs Associated with a Gene list” option allows to select gene(s) from a pull down gene list and fetch the myomiRs regulating these genes (Fig. 2a). The list contains only those genes which are regulated by myomiRs in the database. “miRNAs Associated as Biomarkers” option allows the user to get a ready list of those miRNAs that are being associated/validated as biomarkers in different muscular atrophic conditions (Fig. 2b). Similarly “miRNAs Associated with a Drug” (Fig. 2c), “miRNAs Associated with a GO ID” (Fig. 2d), “miRNAs Associated with a Gene Ontology Term” (Fig. 2e), “miRNAs Associated with a KEGG ID” (Fig. 2f), “miRNAs Associated with a KEGG Pathway” (Fig. 2g) allows user to fetch miRNA based on associated drug, GO ID, GO term, KEGG ID and KEGG pathway respectively. All the above options, provide a resultant list of miRNAs that are hyperlinked to their respective detailed information pages. The list of these miRNAs can be downloaded in Excel /PDF format for further analysis.

The “Search” option allows the user to select a specific single myomiR from a drop down menu and further link it to its detailed information page.

The details of the myomiR are provided in the information page which may be divided into four sections.

#### *Knowledge base*

**Figure 3:**
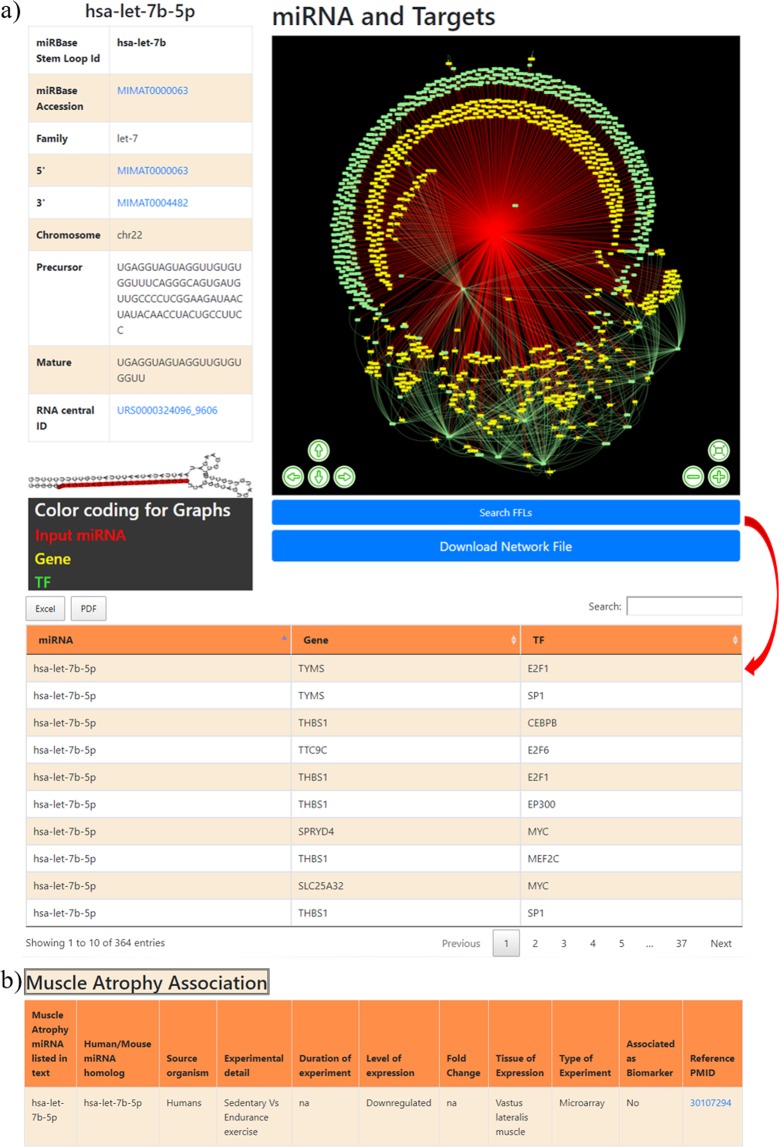
The screenshots of the information page of the myomirDB. (**a**) The web image of the information section of hsa-let-7b-5p. (**b**) The muscle atrophy association of the miRNA is compiled in a tabular format.

This is the first section of the database that gives general information about miRNA i.e. miRBase accession number, 5′/3′ ID, miRNA family, chromosome number, precursor and mature sequences and miRNA stem loop structure. In the stem loop structure, mature miRNA sequence is highlighted in red color (Fig. 3a). These IDs is also hyperlinked to miRBase^23^ and RNAcentral^24^ databases which provide additional details about the miRNA. miRBase database serves as a ready-reference to get details about myomiRs mature and precursor sequence, stem loop structure, chromosome location, source organism etc. Additionally, RNAcentral database provides myomiR transcript genome location, taxonomic details, RNA family classification, miRNA 2D structure etc.

For each miRNA, its target gene and TF targets were identified. Combining this information with TF:gene and TF:miR interactions, a directed miRNA-TF-TG co-regulatory network is constructed for each myomiR in the database. The networks are colour coded (miRNA-red, TF-green, TG-yellow) and interactive which provide zoom and translation options. The network images are high-quality and publication-ready. The interaction network may be downloaded in MITAB 2.5 format for interoperability and can be readily used with many popular visualization tools like Cytoscape^25^, Gephi^26^, BINA^27^ etc. The section also provides an option to download the FFLs in the network.

Biomolecules such as miRNA, gene, transcription factors, etc. often form a recurring pattern in the co-regulatory network that carry out key functions during many pathophysiological conditions. These repeating patterns are termed as network motifs. One of these motifs is the FFL, which recur throughout the co-regulatory networks. FFL is a three- node tri-partite motif, is composed of a miRNA and a TF, one of which regulates the other, both jointly regulating a TG^28^. Recently FFLs have been found to unveil new mechanistic insights behind deregulation of gene expression in different physiological and disease conditions^17,18^. FFL motifs have been proposed as potential diagnostic, prognostics markers and therapeutic targets^29,30^. MyomirDB identifies FFLs using in-house scripts from the miRNA-TF-TG co-regulatory networks of the myomiRs. The FFLs can be accessed by clicking on “Search FFLs” option of miRNA-information page. The example of hsa-let-7b-5p has been shown in Fig. 3a. The table can be downloaded in Excel /PDF format.

#### *Association with muscle atrophy*

For each miRNA, its association with muscular atrophy is compiled in a tabular format. The details are presented as the source organism (organism in which the study was performed), experiment details, duration of the experiment, level of expression (up/down regulated), fold change, tissue of expression, type of experiment (type of experiment to prove the association), reference paper (Fig. 3b). The association of the myomiR as a biomarker is also compiled i.e. if the myomiR is ever experimentally validated to be a biomarker, the entry in the column will be “Yes” otherwise “No”. The reference papers are hyperlinked to PubMed^31^ which allows ready access to the original paper. In this format, the expression changes of a myomiR in different durations, tissues, and atrophy-disease conditions can be easily and quickly explored, compared and analysed.

#### *Association of miRNA with drugs, tissues and other diseases*

**Figure 4:**
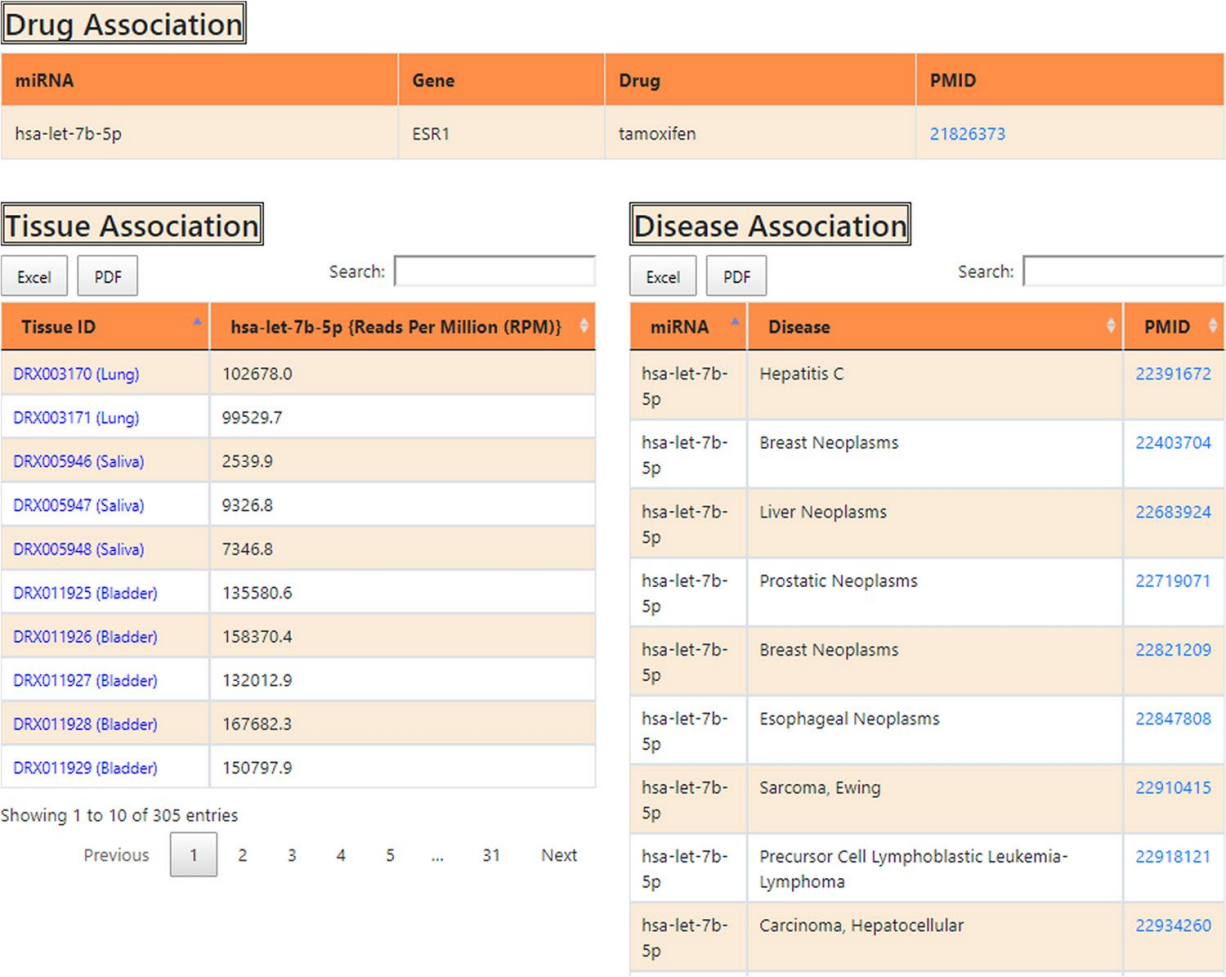
The web image of the third section of the miRNA information page. This section contains miRNA associated information i.e. drug association, expression level in different tissues and cell lines, association with different disease pathologies. The table can be searched through ‘Search’ box and information in table can be downloaded in Excel/PDF format.

This section provides details of drug, tissue, and disease-association of atrophy myomiRs. The information is represented in three tables belonging to each category respectively (Fig. 4). The first table shows information about miRNA gene-target and its associated drug. This type of information can help the users to guide/design any miRNA-based drug-targeting experiment. Similarly, miRNA tissue-specific-expression and miRNA-disease-association tables can be used to identify the potential of the myomiR to be a tissue or a disease-specific marker. The tissue experiment IDs in the tissue association tables are being hyperlinked to the SRA database^32^, which provides complete tissue-specific experimental details. The references for each entry in miRNA-disease-association and miRNA-drug-association tables are also hyperlinked to the PubMed reference paper. These two tables are equipped with the “search” option which help in easy search of user-defined terms across lengthy tables. The tables can also be downloaded in Excel/PDF format.

#### *Association with gene ontologies and pathways*

**Figure 5:**
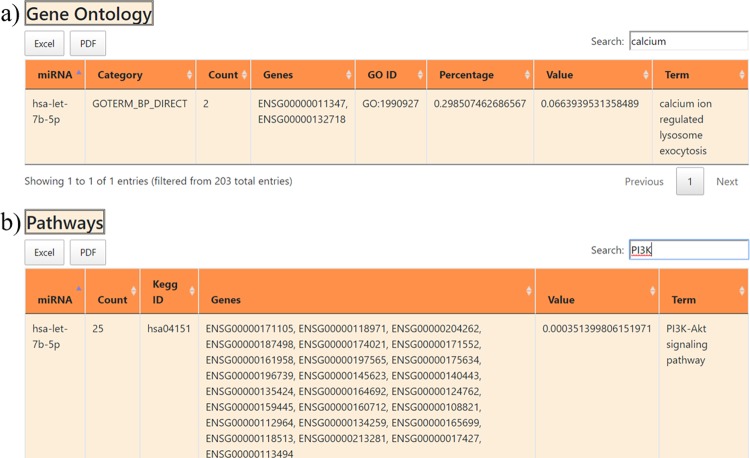
The web images of the fourth section of the miRNA information page. (**a**) The section contains information on enriched GO of miRNA targets (**b**) The section lists the enriched KEGG pathways.

The fourth section of the information-page presents the functional characterization of the miRNA targets. It includes both functional and pathway enrichment that are stored and compiled in tabular format. This section is also provided with a “search” option which allows exploring the extensive tables and fetching the user-defined information using different types of gene/GO-term/KEGG-pathway queries. Eg. The information page of hsa-let-7b-5p contains 203 rows. Figure 5a shows the results if searched with “calcium” keyword to identify specific GO term, which makes browsing the lengthy tables easier. Similarly Fig. 5b shows the results of searching “PI3K” keyword in the KEGG pathways table of hsa-let-7b-5p.

### Webserver statistics

**Figure 6:**
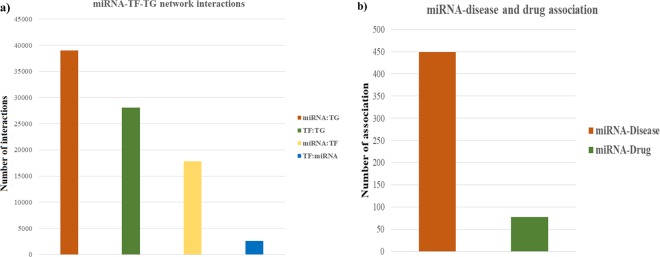
Webserver Statistics (**a**) Number and type of interactions in the database which were used to construct miRNA-TF-TG co regulatory networks (**b**) Number of miRNA to disease and miRNA to drug associations.

MyomirDB is a unique resource that provides a comprehensive and detailed compilation of various bio-molecular interactions; miRNA-TF-TG co-regulatory networks and ~8000 tripartite regulons associated with 247 differentially expressed myomiRs which have been experimentally validated to be associated with various muscular atrophy conditions. 459 muscle atrophy associations of these 247 myomiRs are catalogued. Each database entry contains complete information of the atrophy myomiR, source organism, associated pathophysiological atrophic condition, experiment duration, level of expression (up/down), fold change, tissue of expression, experimental technique for validation, disease and drug association, tissue-specific expression level, Gene Ontology and KEGG pathway associations. The server uniquely constructs miRNA-TF-TG co-regulatory network for each myomiR. For this ~57,000 myomiR: target interactions consisting of ~39,000 miRNA: TG, ~18,000 miRNA:TF interactions were fetched. The current version of MyomirDB has 9,545 target genes and 3,388 target TFs/ TcoFs. Additionally ~28,000 TF:TG and ~2,500 TF: miRNA interactions were fetched and stored in the database (Fig. 6a). The database also contains 449 types of unique miRNA- diseases association and 77 miRNA-drugs association (Fig. 6b).

**Figure 7:**
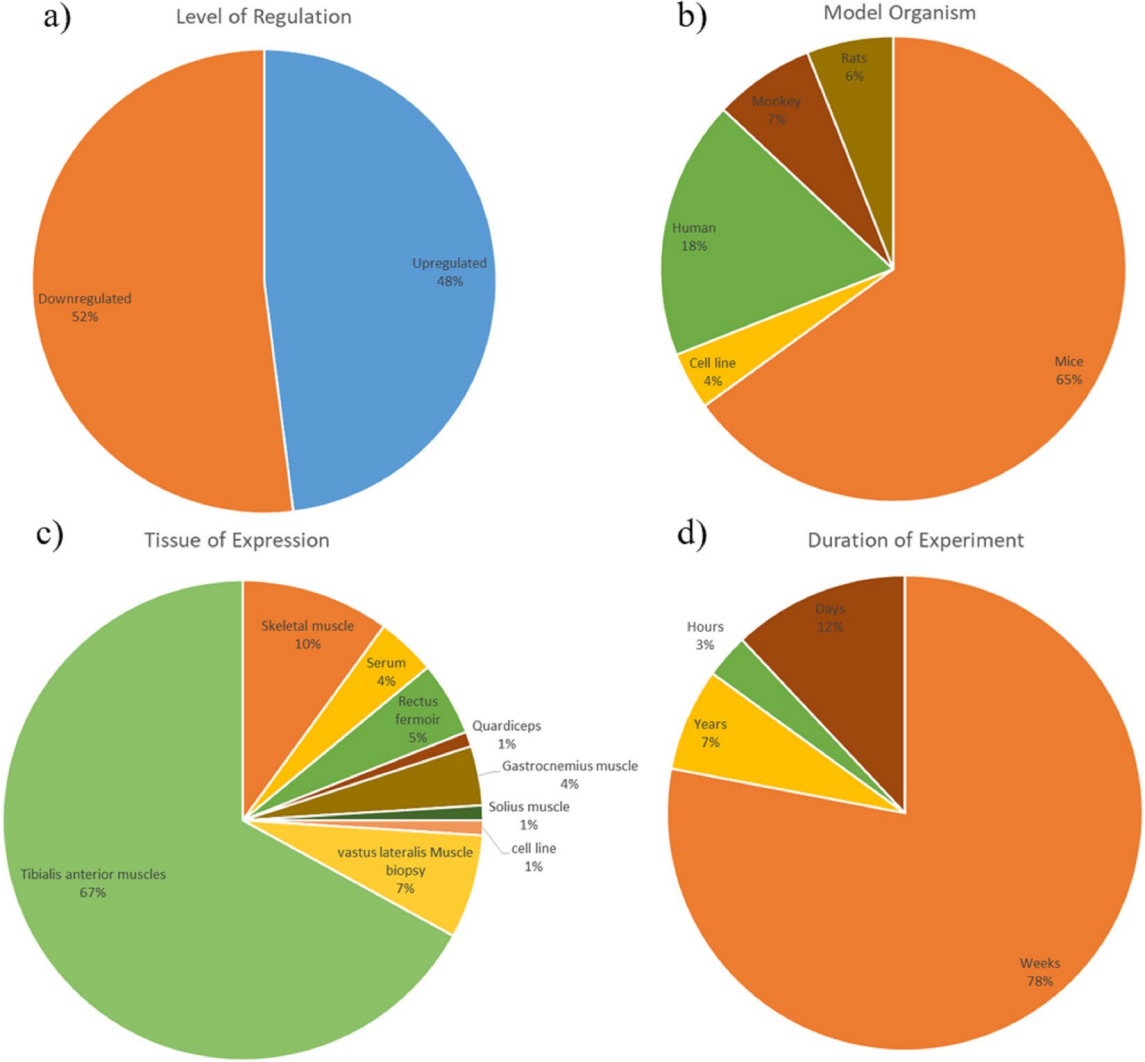
MyomirDB Statistics (**a**) Distribution of myomiRs based on level of regulation (Upregulated and Downregulated) in the database. **(b**) Distribution of myomiRs studied in different source organism. (**c**) Distribution of myomiRs as per their tissue of expression. (**d**) Distribution of myomiRs studied as per duration of experiment.

Out of 247 miRNAs, 52% are downregulated and 48% are upregulated in the database (Fig. 7a). In terms of source organism, 276 database entries were fetched from studies on mouse, 83 from human, 33 from monkey, 33 from rats and 19 from other organism and cell lines (Fig. 7b). In terms of tissue specificity, myomiRs are derived from mainly 9 types of tissues i.e. 67% from tibialis anterior muscles, 10% from skeletal muscle, 7% from vastus lateralis muscle, 5% from rectus fermoir 4% gastrocnemius muscle, 4% from serum and 4% from others tissues (Fig. 7c). The 459 miRNA entries can also be divided into four categories based on the duration of experiment notably, 78% of studies had the duration of experiment ranging in weeks, 12% of studies ranging in days, 3% ranging in hours and 7% were human studies where old age person muscle samples were used for atrophic studies (Fig. 7d).

## Discussion

MyomirDB is an interactive resource and a server platform that captures and organizes knowledge for muscle atrophy myomiRs. MyomirDB provides the comprehensive view of different muscle atrophy myomiRs related studies; offers the annotations and visualization of miRNA- TF-TG co-regulatory network and FFL regulons associated with each myomiR. There are only a few muscle-specific databases like NeuroMuscleDB^20^, SKmDB^21^, MuscleDB^22^ that provides muscle-specific data. NeuroMuscleDB database provides information on genes, mRNAs, and proteins associated with muscle development, neuromuscular diseases, aging, and neurodegeneration but it does not contain any information on miRNAs. SKmDB database contains high throughput NGS datasets to perform integrated sequence analysis but it is specific only to skeletal muscle. MyomirDB is the first of its kind database that has a collection of manually curated differentially expressed muscle atrophy myomiRs that were fetched using text mining and manual curation. The information enables the user to browse muscle atrophy myomiRs based on different query filters in the database i.e. level of expression; tissue of expression; duration of experiment and source organism. MyomirDB also encompasses miRNA-associated information such as miRNA-disease association, miRNA-drug association, miRNA- tissue-specific expression. Hence the information-base of myomirDB is much larger than any other muscle-related databases. MyomirDB also identifies miRNA-target interactions (MTIs) of each myomiR in the database and categories them as miRNA:TF and miRNA:TG interactions. To construct directed tripartite networks, miRNA:TG, miRNA:TF, TF:TG and TF:miRNA interactions were fetched from validated public repositories and added to the database. MyomirDB uses in-house python scripts that utilize simple graph theory for identification of tri-partite miRNA-TF-TG co-regulatory networks and for identification of three-node motif (FFL) pattern in the network. The analysis of miRNA-TF-TG co-regulatory networks of atrophy myomiRs would enable the user to infer mechanistic insights during muscle atrophic conditions. MyomirDB also offers functional correlation of atrophy myomiRs. The functional correlation includes both GO enrichment^33^ and KEGG pathways enrichment^34^. The miRNA associated data can be downloaded from the database in excel/PDF format for further analysis.

### Methodology

#### Data collection

The combination of keywords such “muscle atrophy” and “miRNA”; “muscle wasting” and “miRNA”; “loss of muscle mass” and “miRNA” etc. were used to extract a list 390 publications from PubMed, and Google scholar as in January 2019^31^. After removing redundancy and duplicity, the unique publications were manually curated to identify the Differentially Expressed (DE) miRNAs during muscle-atrophy. For each DE muscle atrophy miRNA, source organism (human or mouse, rat, goat, monkey), atrophy condition (aging, diabetes, cancer etc.), duration of experiment, level of expression (up/down regulation), fold change, tissue of expression, method of experimental validation, association as biomarker and its reference paper was curated from the text. We also recorded whether any literature had associated the miRNA as biomarker during muscle atrophy. Subsequently, miRBase stem Loop Id, miRBase accession number, miRNA Family, 5′ ID, 3′ID, Chromosome number, precursor sequence, mature sequence, and RNA central ID for each myomiRs were retrieved and stored from the miRBase^23^, miRGen 2.0^35^ and RNAcentral^24^ databases respectively. myomiR structure was fetched from miRBase and stored in the database. The mature sequence in the miRNA duplex structure is highlighted in red color.

#### Data processing

Data was processed to maintain uniformity in the nomenclature, the names of precursor miRNAs were mapped to the mature miRNA using miRBase^23^ and miRDB^36^ repository. The updated nomenclature was used and dead/obsolete entries were removed.

For the studies in which the source organism was other than human, the homologous human miRNA was identified. This way, even for experiments conducted on different experimental organisms (mice/rats/goats/rhesus monkey/non-human cell lines**)**, human equivalence/translation would be easier. For some entries, human homologs could not be identified and here mouse homologue were used. Sometimes the information e.g. fold change, tissue of expression were not clearly classified by the author in the publication; that entry would be recorded as ‘na’. If drug and disease associations were not available, the entries are recoded as “na”.

#### Data enrichment

To construct miRNA-TF-TG co-regulatory networks, experimentally-validated gene targets of each miRNA in the database were identified using MirTarBase^37^ and miRecords^38^. A comprehensive list of human TFs and TcoFs was mined from TFcheckpoint^39^, DBD^40^, TcoF-DB V2^41^,TRANSFAC^42^ databases and stored as TF dataset. The gene targets of a miRNA were annotated as a gene or a TF using the TF dataset. The TF:TG interactions were fetched from TRANSFAC^42^, OregAnno^43^ and TRRUST^44^ databases. TF:miRNA interactions were extracted from TransmiR^45^ and PutmiR^46^ databases and stored. The miRNA-TF-TG co-regulatory network of each miRNA in the database was constructed using these interactions. For some mouse miRNAs, human miRNA homologs could not be identified; thus their corresponding targets could not be identified and hence the coregulatory networks could not be built. To make the database informative several other attributes were added; miRNA-disease associations were mined from HMDD^47^; miRNA- tissue association from miRmine^48^ and miRNA–drug relationship from Pharmaco-miR^49^. For each miRNA, GO-functional enrichment^33^ and KEGG-pathway^34^ enrichment of their target genes was performed by Database for Annotation, Visualization and Integrated Discovery (DAVID)^50^ and KEGG mapper respectively. This way, each miRNA was functionally annotated.

### FFL Motif identification

Each vertex in the miRNA-TF-TG coregulatory network is labelled as V_m_, V_TF_ or V_G_. Where, V_m_ refers to a miRNA node, V_TF_ refers to a TF node and V_G_ refers to a gene node. The edges are annotated as E_mt,_ E_mg,_ E_tg,_ E_tm_ where E_mt_ refers to edge from V_m_ to V_TF_, E_mg_ refers to edge from V_m_ to V_G_, E_tg_ refers to edge from V_TF_ to V_G_ and E_tm_ refers to edge from V_TF_ to V_m._

For each V_m_, its edges E_mg_ and E_mt_ were identified from the miRNA:TG interactions and miRNA:TF interactions datasets respectively and the corresponding V_TF_ and V_G_ were listed. Additionally, for each V_m_, its edges E_mg_ and E_tm_ were identified from miRNA:TG interactions and TF:miRNA interactions respectively. Thereafter, a corresponding edge E_tg_ that links V_TF_ to V_G_ is searched in the TF:TG interaction dataset. If found, a tripartite graph containing three vertices (V_m_, V_TF_, V_G_) and edges(E_mt_, E_mg_, E_tg_) was labeled as FFL.

In-house scripts based on this methodology were used to identify FFL motifs for each myomiR in the database.

#### Database development

**Figure 8:**
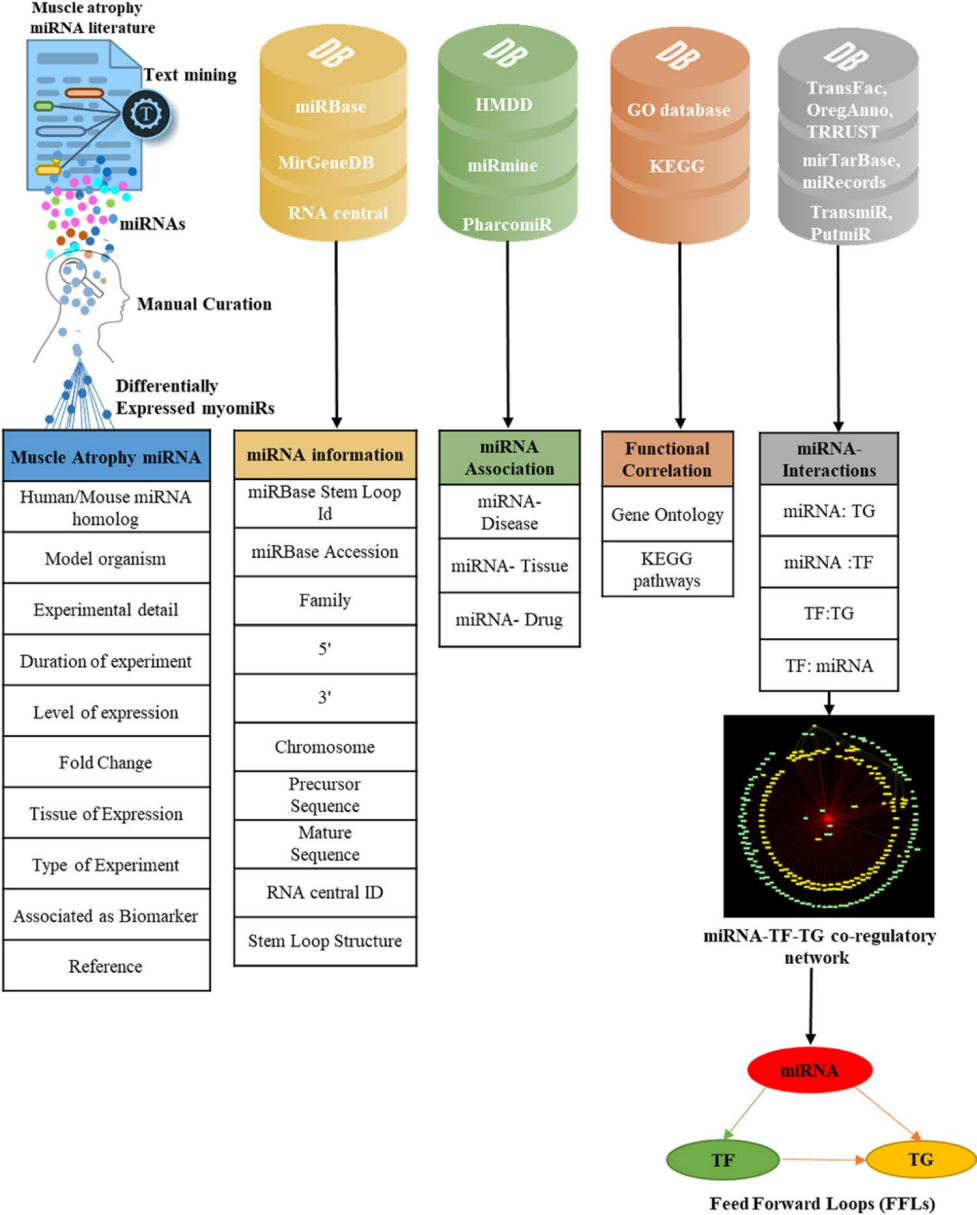
Overview of data collection and annotation in MyomirDB.

All the database files were stored as JavaScript Object Notation (JSON)^51^ files in MongoDB database^52^. These JSON files was uploaded on the server localhost using pymongo and query commands were made in the command line client in MongoDB compass. Figure 8 schematically illustrates the general workflow and features of the resource. Vis.js library specifically was used to display miRNA-TF-TG co-regulatory network at front end^53^. FFLs were identified for each human miRNA by using in-house scripts. The IDs such as miRBase, 5′ ID, 3′ ID, all PubMed references, RNA central ID are hyperlinked to the corresponding databases to provide additional details. The web interface also has a ‘Contact us’ page which includes data submission form for the submission of any new data by the user. It would be reviewed and appended to database.
